# Giant Endometrial Polyp in a Postmenopausal Woman without Hormone/Drug Use and Vaginal Bleeding

**DOI:** 10.1155/2014/518398

**Published:** 2014-06-29

**Authors:** Betül Ünal, Selen Doğan, Fatma Şeyda Karaveli, Tayup Şimşek, Gülgün Erdoğan, Işıl Candaner

**Affiliations:** ^1^Department of Pathology, School of Medicine, Akdeniz University, 07070 Antalya, Turkey; ^2^Department of Obstetrics and Gynecology, School of Medicine, Akdeniz University, 07070 Antalya, Turkey

## Abstract

The objective of this study is to determine and discuss the causes of a giant endometrial polyp in a postmenopausal woman without hormone/drug use and to submit interesting clinical presentation. Here we report a seventy-year-old female patient who was admitted to our hospital with lower back pain. There were no other complaints from her. Physical examination was normal. For further examination, computed tomography was performed and a heterogeneous mass, with a diameter of 10 × 9 centimeters, was detected in the uterine cavity. Hysterectomy because of suspected endometrial cancer was performed. Histopathological examination showed us a giant endometrial polyp with edematous and focal fibrotic stroma, large thick walled blood vessels between normal sized and cystically dilated endometrial glands. To the best of our knowledge, this is the first report of a giant endometrial polyp which is unrelated to use of drugs such as tamoxifen and raloxifene; however, based on the history of the patient it may be associated with long-term consumption of thyme, which is a kind of phytoestrogen.

## 1. Introduction

Endometrial polyps are localized overgrowth of endometrial glands and stroma through the uterine cavity. This benign disease affects 25% of women [1]. They protrude into the endometrial cavity and often have secondary changes. The stroma of the polyp is composed of fibroblast-like spindle cells and large blood vessels with thick walls. The epithelium of the polyp may be active, pseudostratified, or, in postmenopausal period, inactive, flat. Polyps are the common causes of vaginal bleeding in perimenopausal period. However, they are associated with postmenopausal bleeding, infertility, and menorrhagia [2]. Endometrial polyps occur with increased frequency after tamoxifen exposure. They are characteristically multiple, large, and fibrotic. Giant endometrial polyps associated with tamoxifen and raloxifene use were reported in previous studies [3–5].

The prevalence of malignancy with endometrial polyps is 1–3% [6]. The risk factors of malignancy within polyps are ageing, obesity, arterial hypertension, postmenopausal period, and tamoxifen [2]. In addition B. P. Lasmar and R. B. Lasmar [1] reported that endometrial polyps larger than 15 mm are associated with hyperplasia and Wang et al. [7] identified that polyps measuring more than 10 mm are associated with malignancy.

Development of endometrial polyps is affected by unbalanced estrogen therapy, estrogen-like effect, and unbalanced estrogens and progestins. Many estrogen mimics are produced by plants (phytoestrogens (PEs)). PEs are found abundantly in foods, herbs, and spices commonly consumed by humans. It is reported that ER-binding herbal extracts are agonists, much like estradiol; however, PR-binding extracts are neutral or antagonists [8].

Here we presented a giant endometrial polyp in a postmenopausal woman without vaginal bleeding and hormone or drug use. As interesting as her clinical presentation, she had long-term consumption of thyme, which is a kind of PE. Through this rare entity, we discussed the effects of PEs on the female genital tract.

## 2. Case Presentation

A seventy-year-old female patient, G7P7, was admitted to our hospital department of orthopedics with lower back pain. She did not have any other complaints. In the history of the patient, there was only a cholecystectomy history which was performed 26 years ago. Drug use, especially hormone derivatives, was not available, but the patient described consumption of thyme tea by the amount of 1-2 cups a day in the long term, approximately 20–25 years. Orthopedic physical examination was normal; for further examination computed tomography was performed and a heterogeneous mass, with a diameter of 10 × 9 centimeters, was detected in the uterine cavity, whereupon the patient was referred to the department of obstetrics and gynecology. The lesion was asymptomatic and unassociated with vaginal bleeding. Physical examination showed distorted cervix, and because of this preoperative sampling for histopathological diagnosis could not be made. Surgical procedure was planned and hysterectomy because of suspected endometrial cancer was performed. Intraoperative pathology consultation was requested. Accordingly, the mass lesion was reported to be compliant with endometrial polyp, however necessity of multiple sampling was noted.

### 2.1. Gross Evaluation

**Figure 1:**
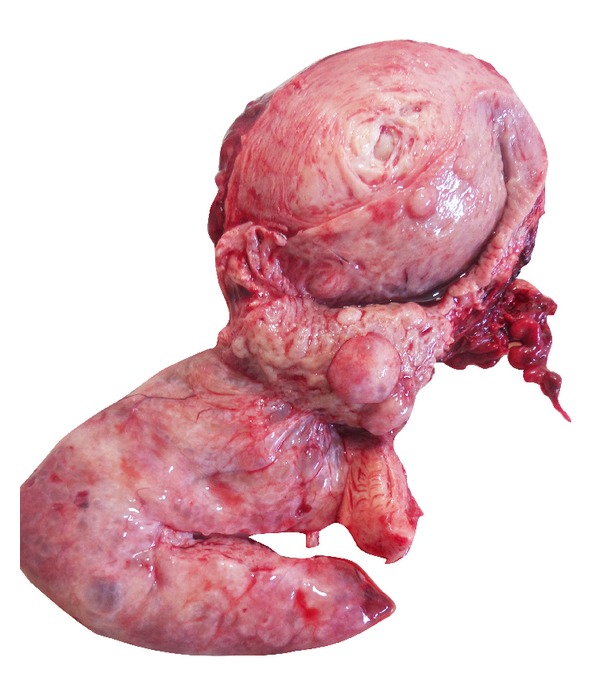
Gross photograph of a giant endometrial polyp in the lower left side of the figure, with smooth surface, cystic changes, and soft consistency. The measurement of the polypoid lesion is 10 × 9.5 × 7 centimeters in diameter.

Macroscopic examination showed us a giant pedunculated polypoid lesion that was extending into the uterine cavity and filling it, with a smooth surface, which is 10 × 9.5 × 7 centimeters in diameter. Cut surface of the polypoid lesion was composed of partially cystic spaces and solid areas, edematous stroma, small foci of fibrous areas, and haemorrhage. The lesion had a soft consistency but it was not degradable (Figure 1).

### 2.2. Microscopic Evaluation

**Figure 2:**
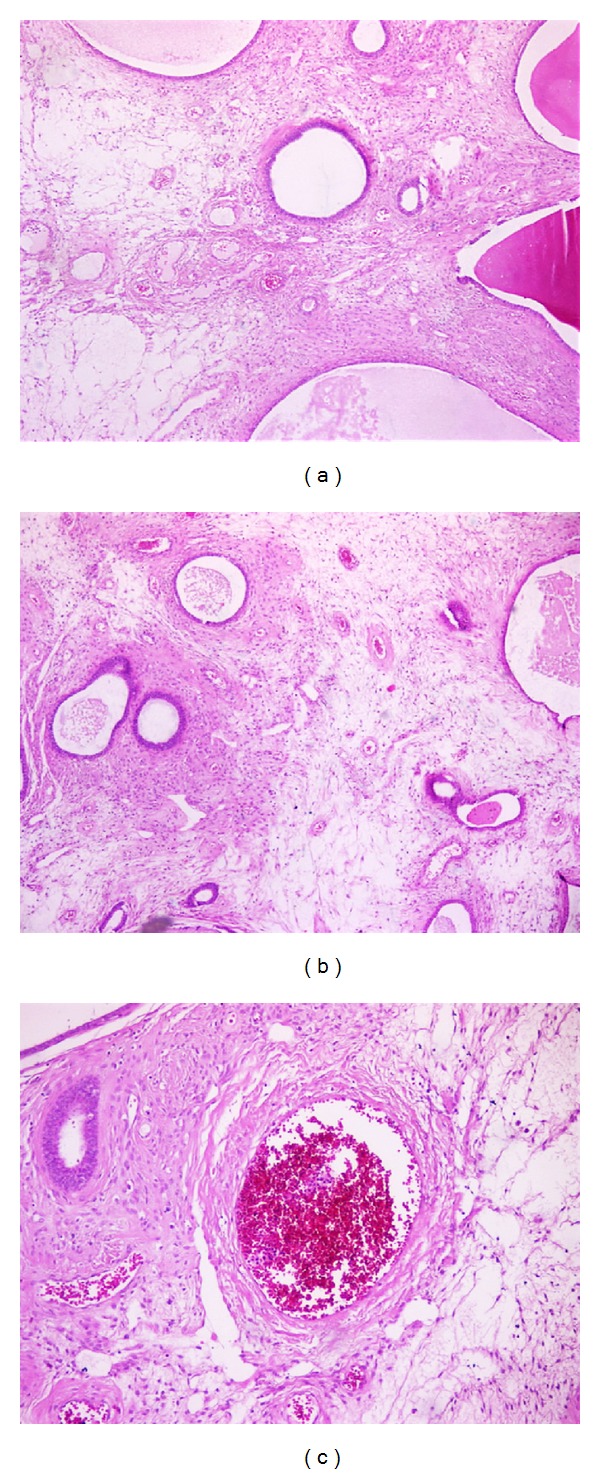
Microscopic examination of the polyp is composed of edematous and focal fibrotic stroma, large thick walled blood vessels between the endometrial glandular structures.

Many sections were taken from the specimen. In microscopic examination, intact large polypoid tissue with cystically dilated glands lined by a single layer of flattened epithelium, large thick walled blood vessels, fibrous stroma with spindled fibroblast-like cells, abundant extracellular connective tissue, and more typical endometrial glands was seen (Figure 2).

## 3. Discussion

Giant endometrial polyps are uncommon variants of classical polyps. Until today only a few cases are reported in the literature and they were associated with tamoxifen and raloxifene treatment [3–5]. Endometrial polyps represent the most common pathology associated with tamoxifen treatment in postmenopausal women with breast cancer, with a prevalence of 13–30% [5]. Tamoxifen is used in the treatment of ER (+) breast cancer. It inhibits the cancer cell growth by the competitive antagonism of estrogen at the estrogen receptor. Despite its role in breast tissue, it is an agonist of estrogen in the endometrium [9].

Indraccolo et al. studied the pathogenesis of endometrial polyps and they demonstrated a causative link: ageing, bcl-2 expression, obesity, tamoxifen regardless of timing, relationship between estrogen receptors and progestins, unbalanced estrogen therapy, estrogen-like effect, and unbalance between estrogen and progestins [10].

PEs are the natural source of estrogen which have been demonstrated by their affinity for the estrogen receptors. PEs are plant-derived compounds that are very similar to estrogen. However, they have shown estrogenic and antiestrogenic activity depending on concentration, endogenous estrogen concentration, and menopausal status. Zava et al. reported that the six highest ER-binding herbs are soy, licorice, red clover, thyme, turmeric, hops, and* Verbena*. In general, they found that ER-binding extracts are agonists, much like estradiol, whereas PR-binding extracts are neutral or antagonists [8]. Endometrial hyperplasia was reported in a study of 179 postmenopausal women after PE administration in the long term (up to 5 years), with a prevalence of 3.37%. PEs have estrogen-like effect and if this effect unbalanced with progesteron, this may be the cause of endometrial hyperplasia [11]. Chandrareddy et al. reported three cases with endometrial pathology to be related to a high intake of PE (soy products). They showed that all three women improved after withdrawal of PE from their diet [12].

Endometrial aromatase activity and gene mutations are associated with endometrial polyp development. However, genistein, which is a kind of PE, can increase aromatase activity in endometrial stromal cells [13].

We reported a case of asymptomatic giant endometrial polyp which was unassociated with drug use, especially hormone derivatives, such as tamoxifen. However, our patient described long-term consumption of thyme which is a kind of PE. She has used the amount of 1-2 cups of thyme tea a day over 20–25 years. We could not detect any other relations with the pathogenesis of giant endometrial polyp. As is known, thyme is a PE and PEs can act like estrogen and this can lead to uterine pathologies, like endometrial polyp. In the literature the effects of PEs were shown in some studies, but the pathophysiology was not fully understood.

In summary, the origin and pathogenesis of endometrial polyps are not fully evaluated. Giant endometrial polyps are rare entities and they are associated with tamoxifen and raloxifene treatment. Endometrial polyp development is depending on estrogen stimulation and unbalanced estrogen. PEs, such as thyme, are plant-derived compounds and they are associated with uterine pathologies. To the best of our knowledge, this is the first report of a giant endometrial polyp which is unrelated to the use of drugs such as tamoxifen and raloxifene; however, based on the history of the patient it may be associated with long-term consumption of thyme, which is a kind of PE.
